# Sustainable Curcumin-Loaded Poly(Glyceryl Adipate) Nanoparticles Attenuate Adjuvant-Induced Arthritis: A Comprehensive Study from Synthesis to Gene Expression Analysis

**DOI:** 10.3390/biotech15020037

**Published:** 2026-05-27

**Authors:** Rania H. H. Ahmed, Mahmoud E. Soliman, Sherif F. Hammad, Hesham S. M. Soliman, Ahmed Abdel-Mawgood

**Affiliations:** 1 Biotechnology Program, Faculty of Basic and Applied Science, Egypt-Japan University of Science and Technology, New Borg El-Arab City 21934, Alexandria, Egypt; rania.hassan118me@gmail.com; 2 Department of Pharmaceutics and Industrial Pharmacy, Ain Shams University, Abbassia 11566, Cairo, Egypt; mahmoud.e.soliman@pharma.asu.edu.eg; 3 Department of Pharmaceutics and Industrial Pharmacy, Faculty of Pharmacy, Egypt-Japan University of Science and Technology (E-JUST), New Borg El-Arab City 21934, Alexandria, Egypt; 4 Pharmaceutical Chemistry Department, Faculty of Pharmacy, Capital University (Formerly Helwan University), Cairo 11795, Cairo, Egypt; sherif.hammad@ejust.edu.eg; 5 Medicinal Chemistry Department, Faculty of Pharmacy, Egypt-Japan University of Science and Technology (E-JUST), New Borg El-Arab City 21934, Alexandria, Egypt; 6 Pharmacognosy Department, Faculty of Pharmacy, Capital University (Formerly Helwan University), Cairo 11795, Cairo, Egypt; hesham.soliman@ejust.edu.eg; 7 Pharmacognosy Department, Faculty of Pharmacy, Egypt-Japan University of Science and Technology (E-JUST), New Borg El-Arab City 21934, Alexandria, Egypt

**Keywords:** curcumin, poly(glyceryl adipate) (PGA), polymeric nanocarriers, intra-articular drug delivery, rheumatoid arthritis, adjuvant-induced arthritis (AIA), in silico molecular docking, pro-inflammatory cytokines, sustained drug release, biocompatibility

## Abstract

Curcumin can behave potently as an anti-inflammatory agent, but unfortunately, it suffers from a detrimental aqueous solubility and a limited bioavailability, which hinders its usage in clinical applications. Formulating a polymeric nanosized biocompatible system can increase the delivered curcumin fraction intra-articularly for inflammatory diseases. Poly(glyceryl adipate) nanoparticles were synthesized using a polycondensation process yielding six different polymers, which was followed by a preparation of different curcumin-loaded formulations using the nanoprecipitation method. The selected formulation exhibited a particle size of 115.8 ± 10.9 nm, a mean PDI of 0.191 ± 0.038, a zeta potential of −22.9 ± 1.5 mV, and an EE of about 95%. This formulation was subjected to physicochemical characterization, including TEM, FTIR, DSC, and an in vitro release study. The stability was assessed in the presence of sodium sulfate at different concentrations, and the long-term stability was assessed through three months of storage. The cytocompatibility was evaluated by an MTT assay on normal and cancerous cell lines, revealing no significant cytotoxicity, and RT-qPCR was done in order to point out the anti-inflammation effect of the produced nanoparticles. Finally, a molecular docking study was held against Bruton’s tyrosine kinase. The formulation’s findings demonstrate the potential of nanoparticles, providing a basis for further in vivo studies targeting arthritis therapy.

## 1. Introduction

Millions worldwide suffer from chronic pain and discomfort from different forms of arthritis, such as rheumatoid arthritis. This hindering condition is characterized by persistent inflammation in the synovial joint, which in turn triggers a cascade of progressive cartilage damage and intense pain, and may cause mobility loss if uncontrolled [1]. Medical practitioners frequently utilize non-steroidal anti-inflammatory drugs (NSAIDs) as a cornerstone of modern treatment regimens or direct corticosteroid injections into the joint, which no doubt have downsides [2].

As a better alternative, current research is paying attention to curcumin, which is the main bioactive compound in turmeric. Its potent anti-inflammatory and antioxidant effects are well-documented with a well-known safety profile, making it a perfect candidate for managing arthritis by modulating key inflammatory pathways [3,4]. The major obstacle for clinical usage of curcumin comes from its physical properties; it has a poor solubility in water (around 600 ng/mL), which causes a low bioavailability and prevents it from reaching therapeutic levels in joints [5,6,7].

To overcome these challenges, nanotechnology-based drug delivery can offer encapsulation for a hydrophobic drug like curcumin, which can enhance its solubility, enhance its stability, and modify its release to be slow and controlled over time [8,9,10]. For management of arthritis, injecting such a formulation directly into the joint space is a smart strategy; it concentrates the therapeutic agent at the inflamed site, which maximizes the local effect while minimizing systemic exposure and side effects. It also offers a much longer therapeutic window compared to standard drug injections [11,12,13].

Nanotechnology-based delivery systems have extensively tested the use of many established biodegradable polymers, e.g., poly(lactic-co-glycolic acid) (PLGA). However, there is a rising focus on developing new biomaterials whose reactants come from sustainable and green sources based on their biocompatibility, biodegradability, and renewable building block precursors. Designing a biodegradable polyester such as poly(glyceryl adipate) provides an innovative intersection of effective drug delivery and green chemistry, pointing out a critical need for sustainability in the biomedical field [14,15,16]. The term sustainable in this context refers to the use of renewable, bio-derived precursors (glycerol and adipic acid) and the employment of a solvent-free, catalyst-free melt polycondensation synthesis, which reduces the environmental impact compared to traditional polymer synthesis methods.

With this in mind, the central aim of our work was to synthesize and evaluate a sustainable nanoparticle system from poly(glyceryl adipate) designed to improve curcumin delivery for intra-articular therapy. In this paper, we highlight the PGA synthesis and the initial formulation of the curcumin-loaded PGA nanoparticles. Studies on physicochemical characterization, the stability, the in vitro release profile, the cell viability and a RT-qPCR analysis of inflammatory markers were done. We also employed molecular docking techniques to explore a novel binding interaction between curcumin and Bruton’s tyrosine kinase (BTK), a key target in arthritis-related inflammation. Given the persistent challenges in achieving sustained local drug delivery and maximizing the therapeutic efficacy for inflammatory joint conditions, exploring advanced nanoparticle-based formulations for anti-inflammatory agents like curcumin presents a promising avenue for improving patient outcomes. The principal finding of this research is that our nanoparticle system dramatically improved the solubility and stability of curcumin, showing significant potential as a potentially safe and promising candidate for further toxicological and clinical evaluation for future arthritis treatments.

## 2. Materials and Methods

### 2.1. Materials

We sourced the adipic acid, glycerol, succinic acid, and curcumin from Sigma-Aldrich Chemical Co., Ltd. (St. Louis, MO, USA), while purchasing the acetone and tetrahydrofuran from Piochem (Giza, Egypt). Every solvent utilized was of analytical grade and employed without further purification. For all experimental procedures, we used deionized water.

### 2.2. Synthesis and Characterization of PGD Polymers

Polyglycerol diacid polymer synthesis and characterization were adapted from Mahmoud et al. [15] and Agach et al. [14]. In a round-bottomed flask, glycerol was first mixed with succinic acid and/or adipic acid in different functional molar ratios (FMRs) of glycerol to diacid in order to obtain six different polymers. The reaction mixture was heated on an oil path above a hotplate set to a temperature below 200 °C under constant magnetic stirring. The flask was connected to a vacuum pump to remove the water produced during the reaction, driving the polycondensation reaction to go forward and aiming to reach to the highest possible Mwt and yield. The reaction was allowed to proceed until the mixture changed to a gel-like state. The flask was then removed from the oil bath and allowed to reach room temperature. The final polymer was weighed to determine the overall reaction yield using Equation (1):

(1) $$Polymer\;Yield\;(\%)\;=\frac{Final\;Weight\;of\;Polymer\;}{Initial\;Total\;Weight\;of\;Reactants}\times \;100\;$$

We characterized the selected PGA1 polymer using 1H (400 MHz, Bruker Biospin GmbH, Ettlingen, Germany) in deuterated acetone, processing the resulting data with TopSpin^®^ software (version 4.1.1, Bruker Biospin GmbH, Ettlingen, Germany). To determine the molecular weight profiles—specifically Mn, Mw, and the polydispersity index (PDI = Mw/Mn)—we employed an Agilent PL-GPC-220 high-temperature chromatography system (Agilent Technologies, Santa Clara, CA, USA). For these GPC measurements, three PLgel Olexis columns (300 × 7.5 mm, Agilent Technologies, Santa Clara, CA, USA) were used with THF as the solvent at a flow rate of 1 mL/min. Each sample was prepared by dissolving 4 mg of PGA in 2 mL of stabilized THF using a PL-SP 260VS system (Agilent Technologies, Santa Clara, CA, USA) at 30 °C, and we calibrated the refractive index detector against polystyrene standards [17]. To check for any remaining starting materials, we used a Shimadzu GCMS-QP2020 (Shimadzu Corporation, Kyoto, Japan) equipped with an Rtx-5MS column (30 m × 0.25 mm i.d., 0.25 μm, Restek Corporation, Bellefonte, PA, USA). Following a known protocol [18], the polymer was combined with ethanolic suberic acid and evaporated under nitrogen; the residue was then derivatized with a mixture of pyridine and N,O-bis(trimethylsilyl)trifluoroacetamide (BSTFA) (Sigma-Aldrich Chemical Co., Ltd., St. Louis, MO, USA) for 15 min at room temperature. After diluting the sample in hexane, it was injected using a 1:15 split ratio at 280 °C. The oven was held at 50 °C for 3 min, ramped at 5 °C/min to 300 °C, and held for a final 10 min. Helium served as the carrier gas (3 mL/min), with the mass spectrometer operating at 70 eV and an ion source temperature of 220 °C [19]. Finally, we identified specific monomers by matching their fragmentation patterns against the NIST database (version 17, National Institute of Standards and Technology, Gaithersburg, MD, USA) and the existing literature.

### 2.3. Preparation of Curcumin-Loaded Nanoparticles

The six polymers obtained were tried in drug loading with three different drug amounts of 0.5, 1 and 1.5 mg to yield 18 different formulations in triplicate, in order to select the best-performing formulation. The curcumin-loaded nanoparticles were prepared using a nanoprecipitation method. First, a weighed amount of 10 mg of the synthesized poly(glyceryl succinate-adipate), poly(glyceryl adipate) or poly(glyceryl succinate) polymer along with the three tested amounts of curcumin were co-dissolved in acetone as the organic solvent to create a clear organic phase. This solution was then injected dropwise into 10 mL of 1% poloxamer (Poloxamer 188, Sigma-Aldrich Chemical Co., Ltd., St. Louis, MO, USA) in deionized water [20] under continuous stirring of 1500 rpm as the anti-solvent. As the acetone evaporated, it left a stable aqueous colloidal dispersion of nanoparticles, which was kept in the fridge until used for any further analyses [21,22,23,24,25].

### 2.4. Characterization of the Curcumin-Loaded Nanoparticle Formulations

#### 2.4.1. Particle Size, Polydispersity Index (PDI), and Zeta Potential

We determined the average particle size, polydispersity index (PDI), and zeta potential of the synthesized formulations through dynamic light scattering (DLS) and checked the electrophoretic mobility using a Malvern Zeta-Sizer Nano-ZS equipped with a 632 nm laser (Malvern Instruments Ltd., Malvern, UK). To prevent multiple scattering effects, we diluted each sample with deionized water prior to the analysis [26].

#### 2.4.2. Entrapment Efficiency (EE%) Measurement

The amount of curcumin successfully entrapped within the nanoparticles was determined indirectly. First, a calibration curve for curcumin was generated by using a series of known concentrations within the range of 1–10 µg/mL in a 1:1 ethanol/water solution and measuring UV–Vis spectrophotometrically with NanoDrop™ 2000C (Thermo Fisher Scientific, Waltham, MA, USA), at λmax = 429 nm [27]. To isolate the nanoparticles, the formulation was centrifuged at 15,000 rpm for 30 min at 4 °C until a pellet formed. The amount of free, un-entrapped curcumin remaining in the clear supernatant was then measured using the pre-established calibration curve [28,29]. The entrapment efficiency was calculated using Equation (2):

(2) $$EE\;(\%)\;=\;\frac{Total\;Drug\;Added\;-\;Free\;Drug\;in\;Supernatant}{Total\;Drug\;Added}\times \;100$$

The DLS results alongside the EE% values were the determinants of the best-performing formulation, which was investigated further, physiochemically and in vitro.

#### 2.4.3. Morphological Analysis by Transmission Electron Microscopy (TEM)

We visualized the surface morphology and overall shape of the optimal F7 formulation using a JEOL JEM-2100F instrument (JEOL Ltd., Tokyo, Japan) operated at 200 kV. A drop of nanoparticle dispersion diluted with deionized water was placed onto a carbon-coated copper grid (Ted Pella, Inc., Redding, CA, USA) and left to dry completely. The grid was then examined under the microscope to identify the morphology of the prepared nanoparticles and to validate the size measurements obtained from DLS.

#### 2.4.4. Fourier-Transform Infrared Spectroscopy (FTIR)

An FTIR analysis was carried out to check for any chemical interactions between the drug and the polymer. The spectra were recorded for pure curcumin, the blank poly(glyceryl adipate) polymer, and the final lyophilized curcumin-loaded polymeric nanoparticles. The three samples were scanned using a Bruker VERTEX 70 spectrometer (Bruker Optics GmbH, Ettlingen, Germany) in the range of 4000–400 cm^−1^ with a resolution of 4 cm^−1^ [16].

#### 2.4.5. Differential Scanning Calorimetry (DSC)

The crystallinity of curcumin inside the nanoparticle core was investigated using DSC. Thermal analyses were performed on pure curcumin, the blank poly(glyceryl adipate) polymer, and the lyophilized curcumin-loaded polymeric formulation. Samples were weighed into sealed aluminum pans and heated within a temperature range from room temperature to 300 °C at a constant rate of 10 °C/min under a 30 mL/min nitrogen flow, using a DSC-60 (Shimadzu Corporation, Kyoto, Japan) [30].

#### 2.4.6. Stability Evaluation

The stability of the formulation was checked in two ways. For short-term storage stability, the nanoparticle dispersion was stored in sealed vials at refrigeration (4 °C) temperature. Changes in the particle size, PDI, zeta potential and entrapment efficiency were monitored every month over a period of 3 months.

Separately, the colloidal stability in the presence of electrolytes was evaluated. Equal volumes of the nanoparticle formulation were mixed with sodium sulfate solutions of increasing molar concentrations. After a 30 min incubation at room temperature, the turbidity of each mixture was measured using a UV–Vis spectrophotometer by NanoDrop™ 2000c, at λmax = 429 nm, to determine the critical coagulation concentration (CCC) [31].

#### 2.4.7. In Vitro Drug Release Study

We investigated the release of curcumin from the nanoparticles using a dialysis bag technique. A 0.5 mL volume of the formulation was loaded into a Spectra/Por^®^ dialysis membrane (12–14 kD MWCO, Spectrum Laboratories Inc., Vancouver, BC, Canada), which was then sealed and fully immersed in the release medium (PBS, pH of 7.4). To ensure that the sink conditions were maintained, we added 0.5% *v*/*v* Tween-80 to the buffer. The addition of 0.5% *v*/*v* Tween-80 (Sigma-Aldrich Chemical Co., Ltd., St. Louis, MO, USA) to the release medium was essential to maintain sink conditions, ensuring that the low aqueous solubility of curcumin did not limit the release rate, thereby more accurately reflecting the dynamic environment of the synovial fluid. The entire setup was maintained at 37 °C with continuous shaking [25]. The samples were analyzed at predetermined time points; each time, 1 mL of the medium was removed, and then 1 mL of fresh medium preequilibrated at 37 °C was re-added. The samples were checked on a UV–Vis spectrophotometer (NanoDrop™ 2000c, Thermo Fisher Scientific, Waltham, MA, USA) at 420 nm to see how much curcumin was released [20].

### 2.5. Cytocompatibility via MTT Assay

To ensure the safety of the formulation, the cytocompatibility of the nanoparticles and the free drug was assessed on 4 different cell lines—the normal BJ-1 cell line and the HCT-116, MCF-7 and HT-29 cancerous cell lines—using an MTT assay. BJ-1 fibroblasts [32,33] and MCF-7 [34] were cultivated in DMEM (Gibco, Thermo Fisher Scientific, Waltham, MA, USA), which we supplemented with 10% FBS (Gibco, Thermo Fisher Scientific, Waltham, MA, USA) and 1% penicillin–streptomycin (Gibco, Thermo Fisher Scientific, Waltham, MA, USA). The cells were cultivated in a humid environment containing 5% CO_2_ at a temperature of 37 °C. Subculturing took place when around 80% confluence was attained, using 0.25% trypsin–EDTA (Gibco, Thermo Fisher Scientific, Waltham, MA, USA). HT-29 cells and HCT-116 [35,36] were cultured in McCoy’s 5A medium (Gibco, Thermo Fisher Scientific, Waltham, MA, USA), which we supplemented with 10% FBS and a cocktail of antibiotics. Incubation was performed at 37 °C with 5% CO_2_, and the cells were detached with trypsin–EDTA when reaching 70–80% confluence. The cells were cultured in 96-multi-well plates (Greiner-Bio one GmbH, Frickenhausen, Germany) and, after 24 h, were exposed to increasing concentrations of 12.5 to 100 µg/mL of the nanoparticles and the free curcumin for [48 h]. After the incubation period, the MTT solution (Sigma-Aldrich Chemical Co., Ltd., St. Louis, MO, USA) was added to each well, and we dissolved the resulting formazan crystals in DMSO, then measured the absorbance with a Bio-Rad microplate reader (Model 680, Bio-Rad Laboratories, Inc., Hercules, CA, USA). To determine cell viability, we calculated the percentage relative to the untreated control group using Equation (3):

(3) $$Cytotoxicity\;\%\;=\;100-\;\frac{A570\;of\;treated\;cells}{A570\;of\;control\;cells}\;\times \;100\%$$

### 2.6. Quantitative Real-Time PCR (RT-qPCR) for Inflammatory Markers

To thoroughly investigate the molecular mechanisms underlying the effects of our tested agents, we quantitatively profiled messenger RNA levels of key inflammatory genes. Specifically, expression of TNFα, IFNγ, IL-6, iNOS, IL-10, Cox2, and NF-κB was assessed in BJ1 cells following a 72 h exposure to IC50 doses of the treatments.

The experimental procedure began with BJ1 cells being treated for 72 h with their respective IC50 concentrations of either nanoparticles (NPs), curcumin, or lipopolysaccharide (LPS) (Sigma-Aldrich Chemical Co., Ltd., St. Louis, MO, USA). The control conditions included untreated cells. After the treatment period, the cells were meticulously collected, and following the manufacturer’s protocol, we extracted the total RNA using the Norgen Biotek Corp. Purification Kit (Thorold, ON, Canada).

For cDNA synthesis, we used 1 μg of the resulting purified RNA per sample in a 20 μL reaction volume, utilizing the QuantiTect Reverse Transcriptase Kit (Qiagen, Hilden, Germany). We then assessed gene expression via quantitative PCR on a Rotor-Gene Q 5-Plex HRM thermal cycler (Qiagen, Hilden, Germany), confirming amplicon specificity with the QuantiTect SYBR-Green PCR Kit (Qiagen, Hilden, Germany) as previously described [37].

The 25 μL qPCR reaction mixture contained 5 μL of diluted cDNA, 12.5 μL of 2× SYBR-Green PCR Master Mix, 2.5 μL of each 10 μM primer, and 2.5 μL of RNase-free water. We analyzed each sample in triplicate (both biological and technical replicates) and included a no-template control (NTC) in every run to monitor for contamination. Data processing was performed using the Rotor-Gene^®^ Q software (version 2.1.0, Qiagen GmbH, Düsseldorf, Germany), identifying threshold cycles (Ct) via the second derivative maximum method. We normalized the expression levels against beta-actin and determined the relative fold-change using the 2^−ΔΔCT^ method.

### 2.7. Molecular Docking Analysis

To investigate how curcumin theoretically interacts with the ATP-binding pocket of its target, we conducted a flexible-ligand molecular docking study using AutoDock Vina (version 1.1.2, Center for Computational Structural Biology, Scripps Research Institute, La Jolla, CA, USA). We retrieved the 3D crystal structure of Bruton’s tyrosine kinase (BTK) from the Protein Data Bank (PDB ID: 8FLL; resolution: 2.15 Å). Before docking, we processed the protein structure by stripping away water molecules and co-crystallized ligands, then added polar hydrogens and assigned Gasteiger charges using AutoDock Tools (version 1.5.6, Center for Computational Structural Biology, Scripps Research Institute, La Jolla, CA, USA). The search space was specifically defined using a grid box of [22 × 22 × 22] Å^3^ centered at the coordinates [x = −24.0, y = 1.0, z = −2.5] to encompass the active kinase pocket. The three-dimensional structure of curcumin was sourced from the PubChem database [CID: 969516], and its energy was minimized using the MMFF94 force field implemented in BIOVIA Discovery Studio Visualizer software, (version 21.1.0 Dassault Systèmes, San Diego, CA, USA) to achieve the most stable conformation. The docking simulation was run (with an exhaustiveness value of 32) to predict the binding poses and calculate the binding energies expressed in kcal/mol, which were then compared to those of the known FDA-approved specifically engineered synthetic drug BTK inhibitor ibrutinib [38] to validate the binding affinity and orientation.

### 2.8. In Vivo Study Design and Ethical Approval

We strictly followed the “Guiding Principle in Care and Use of Animals” (DHEW publication No. (NIH) 80-23) for all animal handling and experimental protocols to ensure full ethical compliance. The study protocol, entitled “Production and Evaluation of Curcumin loaded Polymeric Nanoparticles for Arthritis Control,” received full review and approval from the Egypt–Japan University of Science and Technology (E-JUST) Research Ethics Committee (certificate No: # 251109, date of issue: November 2025). This endorsement confirms the study’s compliance with research ethics standards and norms adopted by E-JUST, as outlined in Chapter 9 (The Code of Ethics) of the E-JUST bylaw. A total of eighteen male albino rats, each with an average weight of 250 g, were utilized for this investigation, housed under standardized laboratory conditions.

#### 2.8.1. Induction of Adjuvant-Induced Monoarthritis (AIA)

Experimental arthritis was induced in the rats following an established protocol by Chung et al. [39]. Briefly, anesthesia was first achieved through an intraperitoneal injection of thiopental sodium (Sigma-Aldrich Chemical Co., Ltd., St. Louis, MO, USA). Subsequently, a 150 µL aliquot of complete Freund’s adjuvant (CFA) (Sigma-Aldrich Chemical Co., Ltd., St. Louis, MO, USA), serving as the arthritis inducer, was meticulously injected into the left (ipsilateral) knee joint of each animal. To provide an internal negative control, the contralateral (right) knee joint received an equivalent volume of sterile normal saline solution. The onset of arthritis was rapid, with visible edema and erythema developing in the left knee joints within hours, typically reaching peak inflammatory swelling by three days post-CFA injection. Daily measurements of knee diameters were performed to monitor the progression of inflammation.

#### 2.8.2. Treatment Regimen and Intra-Articular Injections

The treatment protocols commenced three days following arthritis induction, a point designated as day 0 for the treatment phase. Prior to the initial injection, the knee areas were carefully shaved using a depilatory cream to facilitate precise intra-articular administration and accurate subsequent assessments of inflammation. Eighteen albino rats were systematically divided into three distinct groups, with six animals allocated to each group.

Group I (Positive Control): Animals in this group, serving as the arthritic untreated control, received intra-articular injections of 100 µL of sterile normal saline into the left knee joint.

Group II (Free Curcumin): This group was administered free curcumin at a dose of 5 mg/100 µL via intra-articular injection into the left knee.

Group III (Curcumin-Loaded Polymeric Nanoparticles): Rats in this group received intra-articular injections of curcumin-loaded polymeric nanoparticles at a dose of 50 mg/100 µL into the left knee joint. The specific dose of curcumin-loaded nanoparticles and the equivalent free curcumin was determined based on insights from a modified previous study [40].

All specified treatments were administered on a schedule of days 3, 7, 11, and 15, counting from the initiation of treatment (day 0 post-induction).

#### 2.8.3. Assessment of Knee Inflammation

The anti-inflammatory efficacy of the various treatments was quantitatively assessed by monitoring alterations in the knee joint diameter, a direct indicator of swelling and edema. Measurements of the diameter of the left knee joint for all experimental groups were recorded using a Vernier caliper (Mitutoyo Corporation, Kawasaki, Japan) on days 3, 10, 17 and 24 after the initial arthritis induction. The percentage inhibition of inflammation was subsequently calculated to evaluate the therapeutic effect of each intra-articular injection, utilizing Equation (4):

(4) $$\%\;Inhibition\;of\;Inflammation=\;\frac{\left(Vc\;-\;Vt\right)}{Vc}\;\times \;100$$

Here, Vc indicates the mean diameter of the left knee joint for the positive control group (Group I), while Vt refers to the mean diameter for a treated group at a specific measurement interval.

#### 2.8.4. Histopathological Examinations

At the end of the experimental period, all animals were euthanized via cervical dislocation. The ipsilateral knee joints were harvested and immediately placed in 10% neutral-buffered formalin (Sigma-Aldrich Chemical Co., Ltd., St. Louis, MO, USA) for a 72 h fixation period. To facilitate the sectioning of the osseous components, the fixed samples were decalcified in a suitable ethylenediaminetetraacetic acid (EDTA) solution (Sigma-Aldrich Chemical Co., Ltd., St. Louis, MO, USA) before further processing.

Following decalcification, the tissues were meticulously trimmed and dehydrated through a series of increasing ethanol concentrations. The specimens were then cleared in xylene and embedded in Paraplast tissue media (Leica Biosystems, Richmond, IL, USA) to form solid blocks. Using a rotary microtome (Leica RM2235, Leica Microsystems GmbH, Wetzlar, Germany), thin sections of 4 µm were obtained. These sections were stained with Harris hematoxylin and eosin (H&E) (Sigma-Aldrich Chemical Co., Ltd., St. Louis, MO, USA) to evaluate the general morphological integrity of the bone trabeculae, marrow cavity, and surrounding connective tissues.

A microscopic analysis was conducted using a high-resolution light optical microscope (Leica Microsystems GmbH, Wetzlar, Germany). The qualitative assessment focused on identifying osteolytic changes, trabecular connectivity, and myeloid tissue health. Furthermore, a semi-quantitative histological scoring system was employed by a blinded pathologist to grade the severity of the pathology. Based on established protocols [41], four parameters—cartilage/bone erosion, inflammation, synovial hyperplasia, and pannus formation—were evaluated on a scale of 0 (normal) to 4 (severe). The cumulative scores (0–16) served as a metric for the extent of arthritic damage and the subsequent regenerative efficacy of the nano-treatment across the experimental groups.

### 2.9. Statistical Analysis

We performed all measurements in triplicate (*n* = 3), reporting the results as the mean ± standard deviation (SD). To analyze the stability parameters—including the particle size, polydispersity index, zeta potential, and encapsulation efficiency—we used the GraphPad InStat software (version 3.0, San Diego, CA, USA). We applied a one-way analysis of variance (ANOVA) to evaluate how storage time affected each parameter. In cases where the ANOVA indicated significant differences, we followed up with Tukey–Kramer post hoc tests for multiple comparisons. We considered any results with a *p*-value less than 0.05 to be statistically significant. A sample size of *n* = 6 per group was selected based on a power analysis to ensure sufficient statistical sensitivity to detect a 20% difference in the joint diameter with a power of 80%, while adhering to ethical guidelines for minimizing animal use in preclinical research.

## 3. Results

### 3.1. Synthesis and Characterization of PGD Polymers

**Figure 1:**
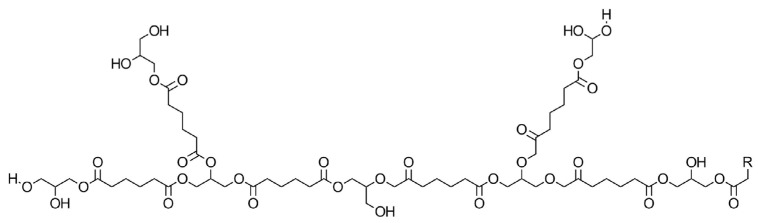
Polyglycerol adipate polyester structure.

The sustainable poly(glyceryl succinate-adipate), poly(glyceryl adipate) and poly(glyceryl succinate) polymers were successfully synthesized via polycondensation, resulting in a consistent yield of around 80%. The chosen polymer, which behaved optimally with the best-performing formula, was polyglycerol adipate polyester, which was synthesized via a melt condensation reaction that did not require a catalyst or solvent, with its structure illustrated in Figure 1.

Glycerol (Gly), adipic acid (ADP), and succinic acid (SUC) are frequently used to synthesize sustainable polyesters, which have a wide range of industrial and technological applications [38,42]. We synthesized PGS (polyglycerol succinate) and PGSA (polyglycerol succinate adipate) polymers using an equal molar ratio of the dicarboxylic acids. To avoid the formation of an insoluble gel, we maintained the reaction under “off-gelation” conditions, utilizing a functional molar ratio (FMR) of 1.7 and 1.5 between the glycerol and dicarboxylic acids. Since this is a melt condensation process, we kept the temperature above the melting points of all starting materials and halted the reaction as soon as the polymer reached a high viscosity to prevent gelation.

**Figure 2:**
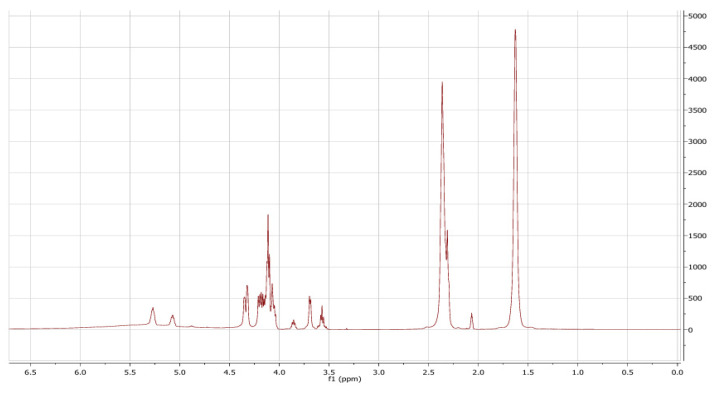
1H NMR spectrum for PGA polymers.

Following synthesis and cooling, we achieved mean yields of 80% ± 0.38 and 73% ± 0.45 for PGA (polyglycerol adipate) with an FMR of 1.5 and 1.7, respectively. To verify the creation of polyester between glycerol and adipic acid, 1H NMR spectroscopy was utilized to determine the existence of distinctive peak integrals in the structure. The 1H NMR spectrum for the PGA polymers in Figure 2 displays its distinct peaks, beginning with those of ADP at δ = 1.65 and 2.4 ppm, corresponding to the 2H of the CH2 group from the adipic end group and adipate ester, respectively [43,44]. Peaks in the range of δ = 3.5–4.5 ppm and 5.0–5.5 ppm were associated with Gly; the latter matched the protons of the recurring units in Gly in the polymer chain, whereas the latter suggested the existence of secondary Gly esters acquired through the polycondensation process [45,46].

**Figure 3:**
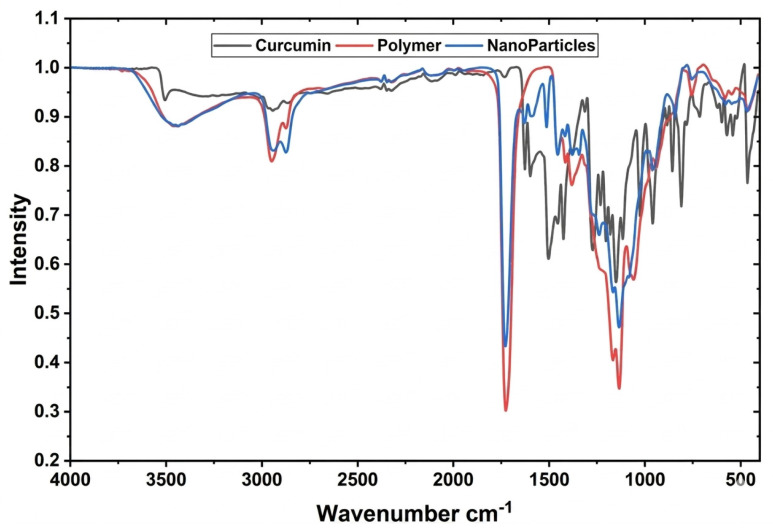
FTIR spectra of: curcumin in grey, PGA in red, and curcumin-loaded nanoparticles in blue.

To confirm the effective esterification of the acid with Gly, the produced polyester was examined through an FTIR analysis. The FT-IR spectroscopy analysis indicated that a polymer possessing a polyester structure was created. As demonstrated in Figure 3, a wide peak was identified between 3200 and 3400 cm^−1^, corresponding to the stretching of the hydroxyl group present in its structure. Peaks observed in the 3000–2900 cm^−1^ range corresponded to the alkyl Csp3-H stretching present in adipic acid. The synthesized polymer exhibited distinct absorption peaks around 1720 and 1140 cm^−1^, the presence of these peaks confirmed the successful formation of the polyester, as the former signal was attributed to carbonyl group stretching and the latter to C-O-C stretching, typically observed in polyesters. The FTIR spectra displayed two peaks around 1720 and 1140 cm^−1^, verifying the successful creation of polyesters [33].

From Table 1, it is clear that, as the FMR rose from 1.5 to 1.7, the average Mw rose as well, suggesting improved esterification and additional elongation of the polymer chains [30,47]. The temperature and FMR were carefully selected while keeping off-gelation conditions to avoid an unintended rise in the MW of the polymers, which could lead to the creation of a gel-like substance with fixed properties that would hinder the development of NPs.

### 3.2. Preparation and Characterization of Curcumin-Loaded Nanoparticles

#### 3.2.1. Particle Size, Polydispersity Index, and Zeta Potential Measurement

As shown in Table 2, eighteen different formulations of curcumin-loaded nanoparticles were created through the nanoprecipitation technique, with each formulation prepared in three copies to verify consistency. After the evaporation of acetone, the dispersions visualized as a stable formulation, showing no clumping or phase separation during the preparation and storage process. Using DLS, the formulations recorded a particle size of 105.6 nm to 561.6 nm, and the polydispersity index (PDI) ranged from 0.048 to 0.777, which is an indication that certain formulations showed greater uniformity compared to other formulations. The zeta potential values were −28.4 to −12.6 mV, indicating that the particles were stable in suspension. The encapsulation efficiency (EE%) remained high in all samples, consistently exceeding 85%, indicating that the polymers effectively loaded curcumin. For the comparison of the systems, we considered the particle size, PDI, zeta potential, and EE%. This analysis revealed that the PGA1 formulation (F9) containing 1.5 mg of curcumin was the optimal choice. This formulation was verified in three distinct batches as well. The measured particle size averaged 115.80 ± 10.91 nm. The PDI mean was 0.191 ± 0.038. The zeta potential recorded was ∑22.9 ± 1.5 mV. In summary, these findings are an indication that PGA1 generated small, relatively uniform, and stable nanoparticles with a high encapsulation efficiency of about 95%, positioning it as the most promising formulation for additional evaluation [48,49].

The 18 formulations detailed in Table 2 resulted from varying the polymer-to-drug mass ratios and polymer type. Formulation F9 was selected as the lead candidate because it achieved the optimal balance of a small particle size (<120 nm) and the highest entrapment efficiency (95%).

#### 3.2.2. Morphology and Size Analysis

**Figure 4:**
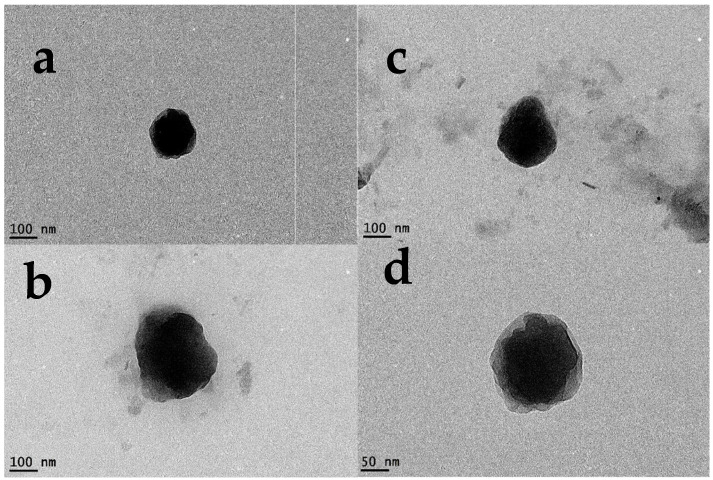
Representative transmission electron microscopy (TEM) images of the selected curcumin-loaded poly(glyceryl adipate) nanoparticles (**a**–**c**) captured at 100 nm and (**d**) captured at 50 nm.

The TEM images in Figure 4 reveal that the particles were distinct, uniformly spherical in shape, and had a solid structure. The particle size observed in the TEM images was around 100 nm, which is in good agreement with the hydrodynamic size measured by the DLS instrument, confirming that the nanoparticles were well-dispersed without significant aggregation.

#### 3.2.3. Fourier-Transform Infrared Spectroscopy (FTIR)

Analyses were performed to confirm that curcumin was successfully loaded into the polymer matrix and to determine its physical state within the nanoparticles. The FTIR spectra for pure curcumin, the blank polymer, and the curcumin-loaded nanoparticles are shown in Figure 4. The FTIR spectra of curcumin showed characteristic peaks at 3500–3200 cm^−1^ corresponding to the phenolic O–H in curcumin and hydroxy groups in the polyglycerol adipate polymer; this peak appeared broadened in nanoparticles, which is an indication that the phenolic O–H groups of curcumin were no longer free. Peaks between 2920 and 2850 cm^−1^ corresponded to C–H stretching in aromatic and aliphatic bonds in curcumin and alkyl C–H stretching in diacids. A strong peak near 1730 cm^−1^ reflected the ester C=O bonds that confirm the successful polyester formation; this peak appeared in the nanoparticle spectrum, which means that the polymer backbone remained intact. The peak between 1625 and 1600 cm^−1^ reflects C=C stretching in the aromatic ring of curcumin, and the band near 1510 cm^−1^ was due to conjugated diketone C=O stretching. C=C and C=O bands appeared with less intensity or were shifted in nanoparticles due to encapsulation. The band near 1150–1100 cm^−1^ indicates C–O–C vibrations in ester linkages [33,50].

#### 3.2.4. Differential Scanning Colorimetry (DSC)

**Figure 5:**
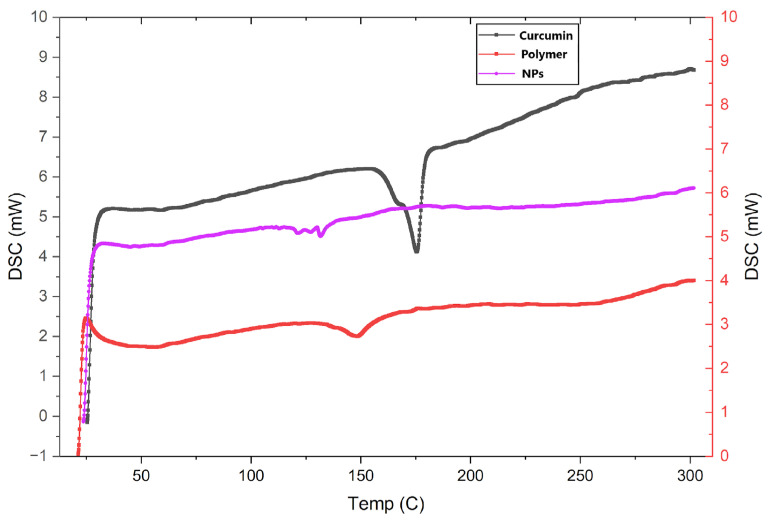
DSC thermograms of pure curcumin, blank polymer, and curcumin-loaded nanoparticles.

The DSC thermograms gave insight into the physical nature of the encapsulated drug, as shown in Figure 5. Pure curcumin exhibited a sharp endothermic peak at 174.3 °C (ΔH = 11.8 J g^−1^), corresponding to its crystalline melting point. In contrast, this peak was completely absent in the thermogram of the final lyophilized nanoparticle formulation. This finding demonstrates that the encapsulated curcumin was no longer in a crystalline state, but was present in an amorphous or molecularly dispersed form within the polymer core. The crystallinity left in the nanoparticles came out to be very low; this supports the idea that the formulation is essentially amorphous [51]. The Tg dropped by about 16 °C, from 147.6 °C to 131.4 °C, in the nanoparticles [52].

### 3.3. In Vitro Curcumin Release Profile

We analyzed how curcumin was released from the nanoparticles using the dialysis setup. The release was followed for 7 days in PBS (pH of 7.4) with 0.5% *v*/*v* Tween-80 to keep the sink condition.

**Figure 6:**
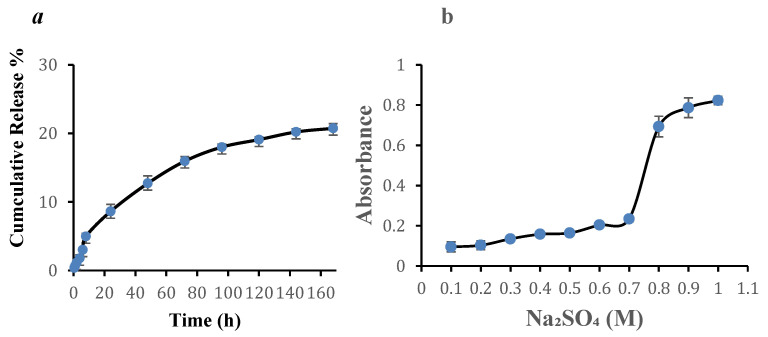
(**a**): In vitro release profile of curcumin from the nanoparticles in PBS (pH of 7.4) + 0.5% *v*/*v* Tween-80 at 37 °C with shaking. Data are presented as mean ± SD (*n* = 3). (**b**): Effect of increasing the sodium sulfate concentration on the turbidity of the nanoparticle formulation.

The curcumin from the nanoparticles showed a slow and controlled release of around 12.72% ± 1.093 in the first 48 h. The release continued gradually with time, until it reached a plateau of 20.74% ± 0.7 by the end of day 7, as represented by Figure 6a.

Overall, the data point to the idea that the nanoparticles provide sustained and controlled release of curcumin and that drug retention was maintained for an extended period, which could reduce the need for multiple dosing and improve patient compliance [25].

### 3.4. Stability Assessment

Firstly, the behavior of the nanoparticles when kept in storage was examined. The samples were stored in closed vials at 4 °C and tested from time to time for about three months. The stability of the formulation was evaluated over a three-month period by monitoring the particle size, polydispersity index (PDI), zeta potential, and encapsulation efficiency (EE%); the detailed numerical values are shown in Table 3.

A one-way ANOVA and subsequent Tukey–Kramer multiple comparisons tests revealed a highly significant effect of storage time on particle size [F(3, 8) = 22.722, *p* = 0.0003]. Particle size exhibited a significant increase from month 2 onwards when compared to day 0, and substantial growth was observed by month 3 across most comparisons, indicating progressive aggregation.

Similarly, a highly significant effect of storage time was observed for the polydispersity index (PDI) [F(3, 8) = 41.590, *p* < 0.0001]. The PDI significantly broadened from month 2 when compared to day 0, with pronounced and significant increases evident by month 3 across multiple time point comparisons, reflecting a growing heterogeneity in the particle size distribution.

For the zeta potential, the one-way ANOVA also indicated an extremely significant effect of storage duration [F(3, 8) = 24.233, *p* = 0.0002]. The absolute negative charge significantly decreased from month 2 onwards compared to day 0, with the most significant reductions observed at month 3 across all earlier time points. This suggests a compromised electrostatic stabilization over time.

Conversely, while a numerical decrease in the encapsulation efficiency (EE%) was observed, the one-way ANOVA showed no statistically significant effects of storage time on the EE% [F(3, 8) = 2.207, *p* = 0.1649]. No significant differences were detected between any time points in the post hoc analyses, suggesting that the observed decline in the EE% did not reach statistical significance under the study conditions.

In summary, the formulation displayed significant physical instability over the three-month period, as evidenced by statistically significant increases in the particle size and PDI, along with a significant reduction in the zeta potential. These changes strongly indicate aggregation and a loss of colloidal integrity. The encapsulation efficiency, however, maintained statistical stability despite a visible trend of decline.

In the second part, the ionic stability of the NPs was tested by a reaction with salt. Different sodium sulfate concentrations were added, and the turbidity was measured UV-spectrophotometrically after 30 min. As shown in Figure 6b, the formulation maintained its stability in low-salt concentrations, with only a slight increase in turbidity. A significant rise in absorbance was easily observed at a concentration of 0.8 M, as the absorbance value was 0.7 ± 0.05, pointing this out as the critical coagulation concentration. This reveals that the nanoparticles are strong enough to withstand moderate ionic strengths, but will aggregate in high-salt environments [31].

### 3.5. In Vitro Cytocompatibility via MTT Assay

**Figure 7:**
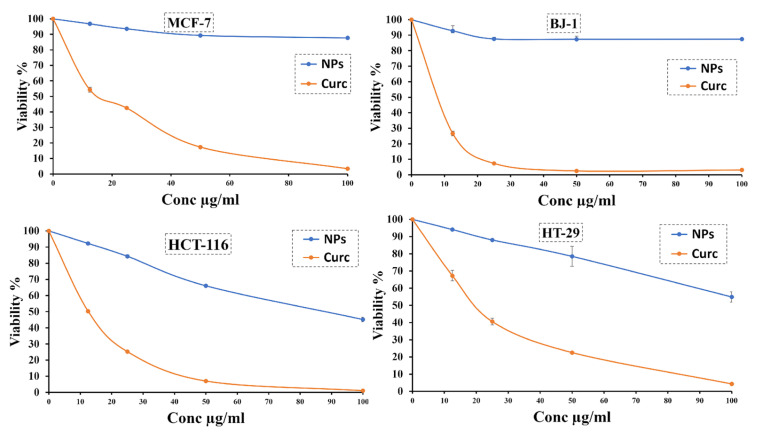
Viability % of free curcumin and NPs in different cell lines.

The impact of free curcumin and the chosen nanoparticles was examined on BJ-1 normal fibroblasts [32,33,53] and the HT-29, HCT-116 and MCF-7 [34,35,36] cancerous cell lines through an MTT assay following 48 h of exposure. As shown by Figure 7, in the normal BJ-1 fibroblasts, there was a very clear difference between the two formulations. Treatment with free curcumin resulted in a noticeable reduction in cell viability across all the concentrations that were tested from 100 µg/mL to 12.5 µg/mL. At the minimum concentration of 12.5 µg/mL, the inhibition was notably high, and based on the dose–response curve, the estimated IC_50_ was under the lowest tested concentration, indicating that the unencapsulated drug demonstrated significant cytotoxicity to normal fibroblast cells without selectivity. Conversely, when the cells were treated with the nanoparticle formulation, the viability values consistently remained high across the tested concentrations, primarily above 85%, even at the highest concentration of 100 µg/mL. The IC_50_ could not be assessed within the concentration range analyzed and was consequently higher than 100 µg/mL. These results suggest that the nanoparticle formulation was mostly cytocompatible with healthy cells and did not induce considerable toxicity, in comparison to the free curcumin, which caused a decrease in cell viability to around 25% even at the lowest concentration used.

In the cancerous cell line, the results showed a more dose-dependent pattern while preserving the fact that the NPs are much safer than the free curcumin, but they may cause more cytotoxicity to cancerous cells than normal cells. This supports the idea that the NPs are selective in their cytotoxicity towards cancerous cells. Free curcumin exhibited strong cytotoxic activity, with inhibition close to 100% at 100 µg/mL, and it still had high cytotoxicity at intermediate doses. From the trend, it can be estimated that the IC_50_ was around 18–20 µg/mL for HT-29, MCF-7 and HTC-116. This confirms that the free drug is potent against this tumor cell line. But the nanoparticle formulation showed a milder cytotoxic profile. The inhibition at the highest concentration was only about 45%, 15% and 55% for HT-29, MCF-7 and HTC-116, respectively.

In general, these results revealed a difference between the two agents. Free curcumin had a greater effectiveness in lowering the viability of different cell lines. The nanoparticle formulation possessed a distinct safety benefit by protecting normal fibroblasts and maintaining their viability nearly unaffected. This specific safety profile indicates that the nano-formulation may be more beneficial for intra-articular use in arthritis, where it is critical to reduce harm to healthy joint tissues while also providing therapeutic effects.

### 3.6. Quantitative Real-Time PCR (RT-PCR) for Inflammatory Markers

To gain insight into the molecular actions of our tested agents, we investigated their impact on the mRNA expression of several key inflammatory genes in BJ1 cells. Our experimental design involved treating BJ1 cells for 72 h with the IC50 concentrations of nanoparticles (NPs), curcumin, or lipopolysaccharide (LPS), with untreated cells serving as a baseline control [37].

**Figure 8:**
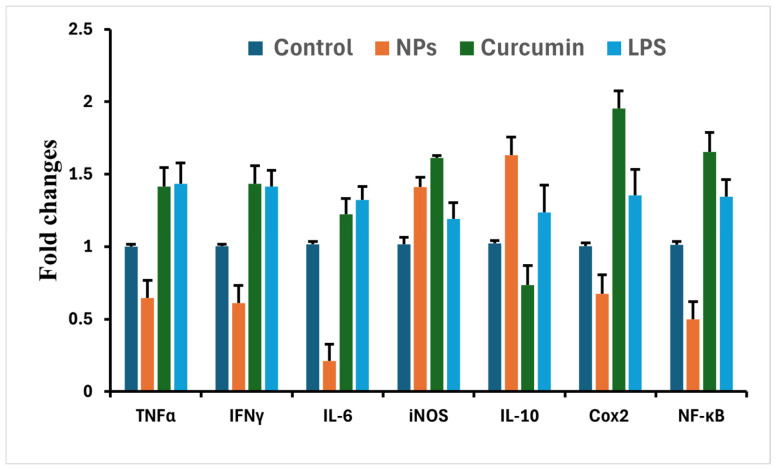
Anti-inflammation effect of control, NPs, curcumin and LPS.

Figure 8 reveals distinct transcriptional responses across the various treatment groups for TNFα, IFNγ, IL-6, iNOS, IL-10, Cox2, and NF-κB. As anticipated, LPS stimulation consistently led to a robust upregulation of all the examined genes, including pro-inflammatory cytokines (TNFα, IFNγ, IL-6), iNOS, Cox2, and NF-κB, as well as the anti-inflammatory cytokine IL-10, thereby initiating a significant inflammatory cascade within the BJ1 cells.

In contrast, treatment with nanoparticles (NPs) generally resulted in a notable suppression of pro-inflammatory gene expression when compared to the LPS-treated group. Specifically, NPs effectively decreased the mRNA levels of TNFα, IFNγ, IL-6, Cox2, and NF-κB. Interestingly, the expression of iNOS and IL-10 did not show a decrease with the NP treatment; instead, their levels were either sustained or slightly elevated. This observation suggests that NPs might play a more complex, potentially dual immunomodulatory role, actively dampening certain pro-inflammatory mediators while either preserving or enhancing other inflammatory or regulatory pathways.

Curcumin, on the other hand, exhibited a broad stimulatory effect on gene expression under these conditions. Curcumin treatment led to an increase across nearly all the assessed genes. The messenger RNA levels for TNFα, IFNγ, IL-6, iNOS, Cox2, and NF-κB were all elevated. The sole exception was IL-10, which did not demonstrate a consistent increase following curcumin treatment. This widespread upregulation across the majority of the inflammatory mediators suggests that curcumin, within our experimental framework, may initiate a distinct cellular response potentially involving different signaling cascades compared to those influenced by NPs.

In summary, the gene expression analysis in BJ1 cells highlighted divergent immunomodulatory profiles. Nanoparticles primarily exerted an anti-inflammatory influence by downregulating most pro-inflammatory markers, whereas curcumin largely induced a broad upregulation of inflammatory mediators. These findings underscore the differential impact of NPs and curcumin on inflammatory gene regulation within the BJ1 cell model.

### 3.7. Molecular Docking Analysis

To provide a theoretical basis for curcumin’s anti-arthritic activity, molecular docking was performed against Bruton’s tyrosine kinase (BTK), a key enzyme in inflammatory signaling. The docking simulations generated ten possible binding poses for curcumin and ibrutinib, the FDA-approved BTK inhibitor, as shown in Table 4.

Upon analyzing the poses, we selected pose number 2 for further inspection due to its strong binding energy, which was comparable to pose number 1 of ibrutinib’s binding energy.

**Figure 9:**
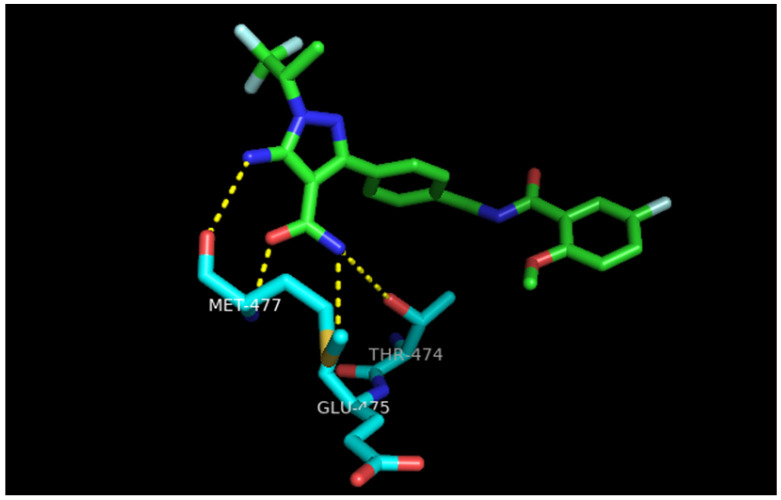
Interaction of ibrutinib with Bruton’s tyrosine kinase.

In this pose, curcumin fits into the active site, forming two critical hydrogen bonds with the key amino acid residues Thr474 and Glu475. These are the same critical residues that the potent inhibitor ibrutinib interacts with in Figure 9, providing a strong rationale for how curcumin can effectively inhibit BTK activity at a molecular level and suggesting a strong binding affinity [38].

To validate the docking procedure, the co-crystallized ligand (ibrutinib) was redocked into the BTK active site. The resulting root mean square deviation (RMSD) was found to be <2.0 Å, confirming the reliability of the binding pose predictions.

### 3.8. Assessment of Knee Joint Swelling

The successful induction of monoarthritis via CFA injection was consistently evident across all animal groups, characterized by the development of significant edema and redness in the left knee joints within hours, progressing to peak inflammation by day 3 post-induction. Subsequent measurements of the knee diameter allowed for the quantitative assessment of anti-inflammatory effects, expressed as the percent inhibition of inflammation relative to the untreated positive control group.

**Figure 10:**
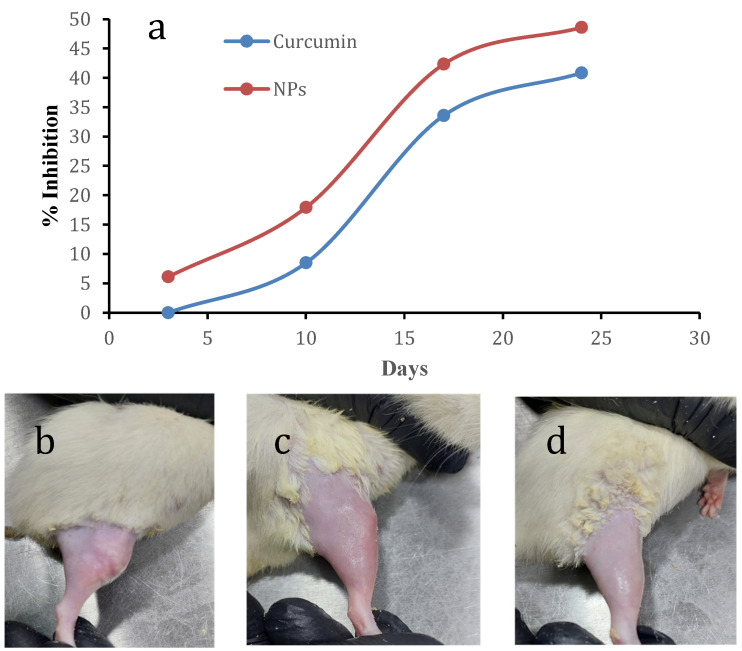
(**a**): The % inhibition of inflammation versus time profile of free curcumin and curcumin-loaded nanoparticles. (**b**–**d**): Macroscopic appearance of ipsilateral (left) rat knee joints: Group I, Group II, and Group III on day 24 post-arthritis induction, respectively.

The summarized data in Figure 10a illustrate the time-dependent changes in the percentage inhibition of inflammation for both free curcumin and curcumin-loaded polymeric nanoparticles (NPs).

On day 3, the free-curcumin group displayed no measurable inhibition of inflammation (0%), whereas the curcumin-loaded NP group demonstrated a modest initial inhibitory effect of 6.10%. Progressing to day 10, both treatment groups exhibited an observable reduction in inflammation; the free-curcumin group showed 8.49% inhibition, while the curcumin-loaded NP group achieved a more pronounced 17.92% inhibition. This pattern of superior anti-inflammatory activity in the nanoparticle-treated group persisted throughout the study duration. By day 17, the percentage inhibition for free curcumin had increased to 33.58%, whereas curcumin-loaded NPs reached 42.34%. At the final assessment point on day 24, free curcumin yielded a 40.85% inhibition, with the curcumin-loaded NPs demonstrating the highest anti-inflammatory effect at 48.59%.

At the conclusion of the study on day 24, visible differences in the knee joint morphology were prominent among the treatment groups, as illustrated in Figure 10. Figure 10b shows a representative ipsilateral (left) knee joint from the positive control group (Group I), clearly exhibiting persistent and severe swelling, redness, and overall inflammation. In contrast, the left knee joint from the free-curcumin-treated group (Group II), depicted in Figure 10c, demonstrated a noticeable reduction in macroscopic inflammatory signs, though some residual swelling was still apparent. Most strikingly, the knee joint from the curcumin-loaded polymeric nanoparticle-treated group (Group III), presented in Figure 10d, displayed the most significant visual amelioration of inflammation, appearing much closer to a healthy, non-inflamed state with minimal swelling or erythema. These macroscopic observations provided the initial visual evidence of the differential therapeutic effects of the administered treatments.

These findings collectively indicate a sustained and notably more effective reduction in knee joint swelling with the curcumin-loaded polymeric nanoparticles when compared to the administration of free curcumin.

### 3.9. Histopathological Findings

Histopathological examinations of the bone tissues across all experimental groups were performed to evaluate the regenerative effects of the administered treatments on the bone architecture and marrow integrity.

**Figure 11:**
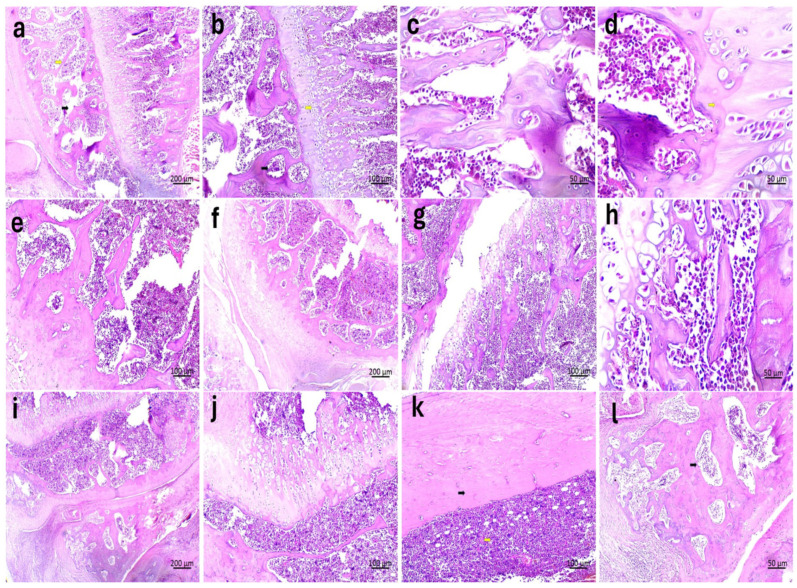
Comparative histopathological analysis of bone tissue across experimental groups: Group 1 (**a**–**d**), illustrating advanced degenerative changes and osteolysis; Group 2 (**e**–**h**), showing early trabecular repair and cellular infiltration; and Group 3 (**i**–**l**), demonstrating the restoration of a normal physiological architecture and cortical integrity (H&E stain). [Key to symbols: Yellow arrows indicate osteolysis/bone loss (**a**–**d**) and healthy repopulated stem cells (**k**,**l**). Black arrows indicate ischemic necrosis/reduced marrow cells (**a**–**d**) and normal Haversian canals (**k**,**l**)].

Looking at the specimens in Group 1, the most striking feature is the level of structural decay. In Figure 11a,b, we can see that the bone trabeculae have thinned significantly, showing a jagged irregularity that points to active osteolysis [indicated by yellow arrows]. There is also clear evidence schemic necrosis within the myeloid tissue—this, combined with the vacuolation seen in the marrow, suggests that the environment has been severely compromised [indicated by black arrows]. By the time we reach Figure 11c,d, the intertrabecular spaces have widened so much that the bone density appears collapsed [with prominent osteolysis shown by yellow arrows and reduced marrow cells by black arrows]. The trabeculae here are delicate, almost fragmented, with osteoporotic cavities and erosion sites visible throughout the tissue.

In Group 2, the bone tissue starts showing the first signs of recovery, even though some underlying damage is still visible. Looking at Figure 11e,f, we can see that, while the trabeculae remain relatively thin, they are starting to reconnect and show active bone formation. There is also a noticeable shift in the marrow spaces; they are not as empty anymore and show a much higher density of cells. By the time we get to Figure 11g,h, the trabecular structures have thickened significantly. They still look a bit fragmented in places, and some erosion is visible, but the narrow marrow spaces are now clearly filled with active stem cells. This transition away from the necrotic state seen in Group 1 is quite distinct.

The specimens in Group 3 represent a near-complete restoration of the bone’s physiological structure. In Figure 11i,j, there is a robust increase in bone density and marrow activity, with the presence of proteinaceous fluid supporting a healthy cellular environment. The trabecular network here is well-connected and shows strong evidence of active formation. The structural integrity is most obvious in Figure 11k,l. Here, the bone has returned to its typical architecture, featuring a solid cortical shell over a healthy network of cancellous bone. You can clearly identify the Haversian canals [black arrow] and osteocytes sitting within their lacunae. The periosteum and endosteum appear regular, facing a marrow cavity that is fully populated with stem cells [yellow arrow], indicating that the tissue has returned to a homeostatic state.

These findings confirm the superiority of the nano-treatment in promoting bone healing and structural consolidation, effectively reversing the osteolytic damage observed in the untreated arthritic/bone-loss model. Despite the positive histological outcomes, this study has limitations, including a relatively small sample size (*n* = 6), lack of comparison with standard treatments, and a lack of pharmacokinetic/biodistribution data. Future studies should quantify the retention time of the nanoparticles within the joint and assess potential systemic leakage.

## 4. Discussion

In this study, we successfully developed a reproducible drug delivery platform using a sustainable, green polymer—poly(glyceryl adipate)—to enhance the therapeutic potential of curcumin for intra-articular arthritis treatment. The overall findings demonstrate that our nanoparticle formulation not only overcomes curcumin’s inherent physicochemical limitations, but also represents a safe and effective system for localized drug delivery.

This work allowed us to precisely control the nanoparticle characteristics, leading to a final formulation with a particle size of approximately 115.8 ± 10.9 nm, a mean PDI of 0.191 ± 0.038, and a zeta potential of −22.9 ± 1.5 mV. A small particle size is critically important for intra-articular applications, as it has been shown to improve tissue penetration and retention within the synovial joint, while a low PDI indicates a uniform and stable system, and exhibiting this range of zeta potential provides sufficient electrostatic repulsion to prevent particle aggregation and ensure good colloidal stability [48]. The high colloidal stability observed in the selected nanoparticle formulation can be explained by the synergy between electrostatic and steric stabilization. The negative zeta potential (approximately −22 mV) provides significant electrostatic repulsion between the nanoparticles, preventing them from clumping. This is complemented by hydrophilic interactions between the poloxamer 188 coating and the aqueous environment. The hydrophilic chains of the surfactant form a hydrated layer on the particle surface, providing a steric barrier that further prevents aggregation even in the presence of electrolytes.

One of the most significant outcomes of our study is the remarkably high entrapment efficiency of over 95% for curcumin. This is a substantial improvement over many previously reported curcumin delivery systems, which often struggle with lower loading capacities [49]. We attribute this success to the inherent compatibility between the hydrophobic curcumin and the polyester matrix, as well as the nanoprecipitation method, which rapidly traps the drug within the forming polymer core. The DSC analysis confirmed our hypothesis that curcumin was molecularly dispersed within the nanoparticles, as evidenced by the disappearance of its crystalline melting peak.

The in vitro release study revealed a sustained release pattern, which is ideal for arthritis therapy. The extended release of curcumin provides long-term control over the inflammation, reducing the need for frequent injections and improving patient compliance. This controlled release profile is a clear advantage over the rapid clearance of standard intra-articular injections [30].

From a clinical translation perspective, safety and biocompatibility are paramount. Our MTT assay results were highly encouraging, showing no significant cytotoxicity of the poly(glyceryl adipate) nanoparticles against a normal cell line and selectivity towards cancerous cells. This confirms the inherent biocompatibility of the glycerol-based polyester, supporting its potential for safe use in intra-articular applications, where direct contact with synovial cells and cartilage is unavoidable [54].

The use of BJ-1 human foreskin fibroblasts as a model allows for the assessment of anti-inflammatory effects in a non-cancerous human lineage. While the results show significant downregulation of pro-inflammatory markers (TNFα, IL-6), it is important to note that cell culture models lack the complexity of the whole joint organ system, and the findings should be correlated with the in vivo data.

Furthermore, our molecular docking analysis provided valuable mechanistic insight into curcumin’s therapeutic potential. The strong binding affinity and, more importantly, the specific hydrogen bond interactions with the critical residues Glu475 and Thr474 within the BTK active site provide a compelling molecular rationale for its anti-inflammatory effects. By showing that curcumin can engage with the same key residues as the potent clinical inhibitor ibrutinib, we strengthen the argument for its use as a BTK-targeting agent in inflammatory arthritis. Our nanoparticle system, by delivering curcumin effectively to the joint space, could enable it to reach the necessary concentrations to engage this target and exert a meaningful therapeutic effect.

Looking forward, while the in vivo results are promising and show the superior and sustained anti-inflammatory effects of curcumin-loaded polymeric nanoparticles, both in terms of knee swelling reduction and histological preservation, which strongly support the hypothesis that targeted, controlled release strategies can overcome the limitations associated with traditional free drug administration in joint pathologies, the next logical step is to validate these findings by gene expression studies performed on tissues from the treated joints to confirm that the delivered curcumin is indeed modulating the BTK signaling pathway and other inflammatory markers as predicted by our docking studies.

Regarding the clinical potential of the system, the biocompatibility of the PGA carrier is supported by its degradation into endogenous, non-toxic metabolites (glycerol and adipic acid), which minimizes the risk of a chronic inflammatory response within the synovium. Furthermore, the intra-articular route of administration was strategically chosen to bypass the formidable barriers of a poor systemic bioavailability associated with oral curcumin. This localized delivery ensures that a high therapeutic payload is maintained directly at the site of joint pathology while drastically reducing the risk of systemic side effects.

Finally, the sustainable poly(glyceryl adipate) nanoparticles developed in this work represent a significant step forward in the delivery of curcumin. We successfully formulated a stable, biocompatible system that dramatically improves curcumin’s solubility and provides controlled release. These findings strongly support the potential of this green nanomedicine platform as a sustainable, reproducible and effective strategy for the localized treatment of arthritis.

## 5. Conclusions

In this study, a sustainable nanoparticle system based on poly(glyceryl adipate) was successfully developed and characterized for the enhanced delivery of curcumin. The selected formulation demonstrated excellent physicochemical properties, including a small, uniform particle size ideal for intra-articular administration, an exceptionally high entrapment efficiency, and a sustained release profile suitable for prolonged anti-inflammatory action.

Crucially, this nano-formulation transformed curcumin into a stable, amorphous, and aqueous-dispersible form, overcoming its primary limitation of a poor solubility. The nanoparticles were confirmed to be highly cytocompatible, underscoring the safety of this green polymer for biomedical use. These experimental findings, supported by molecular docking results that reinforce curcumin’s potential to inhibit the key inflammatory target BTK, establish a solid foundation for this platform’s therapeutic application.

Ultimately, this study underscores the potential of curcumin-loaded polymeric nanoparticles as a viable and effective strategy for managing inflammatory arthritis, paving the way for future translational research into enhanced intra-articular therapies. This study clearly demonstrates the significant potential of poly(glyceryl adipate) curcumin nanoparticles as a next-generation strategy for the effective, localized treatment of inflammatory joint diseases.

## Figures and Tables

**Table 1:** The Mw analysis of PGA obtained by the GPC along with the reaction yield.

Polymers	FMR	Mn (g/mol)	Mw (g/mol)	PDI	Yield%	Consistency
PGA1	1.5	1034	1176	1.13	80 ± 0.38	Viscous
PGA2	1.7	2378	3480	1.46	73 ± 0.45	Viscous

Mn: number average molecular weight, Mw: mass average molecular weight, PDI: molecular weight polydispersity index, and FMR: Gly/adipic functional molar ratio.

**Table 2:** DLS and ELS data of the 18 different nano-formulations produced.

Formula ID	Size (nm) ± SD	PDI ± SD	Zeta Potential (mV) ± SD
F1	204.57 ± 44.35	0.401 ± 0.065	−18.57 ± 4.01
F2	391.03 ± 115.91	0.449 ± 0.055	−19.90 ± 2.25
F3	400.50 ± 31.04	0.425 ± 0.033	−18.57 ± 4.12
F4	209.50 ± 57.19	0.506 ± 0.115	−21.03 ± 2.61
F5	447.07 ± 101.87	0.626 ± 0.144	−21.57 ± 1.89
F6	534.10 ± 387.82	0.545 ± 0.203	−24.60 ± 1.21
F7	142.40 ± 16.80	0.098 ± 0.051	−20.70 ± 0.52
F8	162.47 ± 70.26	0.125 ± 0.014	−22.53 ± 0.06
F9	115.80 ± 10.91	0.191 ± 0.039	−22.87 ± 1.50
F10	239.10 ± 40.38	0.214 ± 0.049	−18.60 ± 3.31
F11	231.37 ± 28.99	0.228 ± 0.064	−21.53 ± 6.05
F12	285.57 ± 110.87	0.220 ± 0.109	−17.13 ± 2.92
F13	263.50 ± 81.39	0.301 ± 0.106	−18.30 ± 4.88
F14	303.33 ± 118.31	0.351 ± 0.012	−18.97 ± 5.55
F15	340.20 ± 152.70	0.402 ± 0.115	−15.20 ± 3.36
F16	169.20 ± 27.98	0.205 ± 0.011	−18.33 ± 1.51
F17	176.60 ± 14.24	0.321 ± 0.135	−20.77 ± 1.96
F18	174.13 ± 11.21	0.251 ± 0.014	−25.57 ± 4.00

**Table 3:** Effect of short-term storage at 4 °C on the nanoparticle formulation.

Time Point	Particle Size (nm) (Mean ± SD)	PDI (Mean ± SD)	Zeta Potential (mV) (Mean ± SD)	EE (%) (Mean ± SD)
Day 0	115.8 ± 10.9	0.191 ± 0.038	−22.9 ± 1.5	95.6 ± 1.1
Month 1	122.2 ± 0.8	0.225 ± 0.012	−21.8 ± 1.21	94.3 ± 2.0
Month 2	144.1 ± 7.5	0.283 ± 0.043	−20.03 ± 0.63	92.1 ± 3.1
Month 3	172.7 ± 13.16	0.461 ± 0.027	−16.2 ± 0.4	88.2 ± 6.5

**Table 4:** Ten different poses of ibrutinib with BTK and curcumin with BTK.

Mode	Ibrutonib Affinity (kcal/mol)	RMSD l.b.	RMSD u.b.	Curcumin Affinity (kcal/mol)	RMSD l.b.2	RMSD u.b.3
1	−11.1	0	0	−8.7	0	0
2	−10.3	1.491	2.067	−8.5	1.822	2.892
3	−10	2.786	4.08	−8.3	1.566	2.157
4	−10	1.227	1.672	−8.3	1.748	11.209
5	−9.9	2.275	3.061	−8.2	1.72	2.673
6	−9.7	2.884	4.569	−7.9	3.144	9.456
7	−9.7	3.498	4.923	−7.7	3.863	7.366
8	−9.6	2.842	4.019	−7.7	2.223	10.441
9	−9.4	2.41	10.356	−7.7	1.934	3.063
10	−9.4	2.477	10.224	−7.6	1.631	11.734

## Data Availability

The original contributions presented in this study are included in the article. Further inquiries can be directed to the corresponding author.

## Abbreviations

The following abbreviations are used in this manuscript:

PDI	Polydispersity Index
DLS	Dynamic Light Scattering
TEM	Transmission Electron Microscopy
FTIR	Fourier-Transform Infrared Spectroscopy
DSC	Differential Scanning Calorimetry
EE%	Entrapment Efficiency
Curc	Curcumin
BTK	Bruton’s Tyrosine Kinase
CCC	Critical Coagulation Concentration
MTT	3-(4,5-Dimethylthiazol-2-yl)-2,5-Diphenyltetrazolium Bromide
MWCO	Molecular Weight Cut-Off
PBS	Phosphate-Buffered Saline
NSAID	Non-Steroidal Anti-Inflammatory Drug
DMEM	Dulbecco’s Modified Eagle Medium
FBS	Fetal Bovine Serum
FMR	Functional Molar Ratio
PDB	Protein Data Bank
